# The Impact of Opportunistic Infections on Clinical Outcome and Healthcare Resource Uses for Adult T Cell Leukaemia

**DOI:** 10.1371/journal.pone.0135042

**Published:** 2015-08-14

**Authors:** Toshiki Maeda, Akira Babazono, Takumi Nishi, Midori Yasui, Shinya Matsuda, Kiyohide Fushimi, Kenji Fujimori

**Affiliations:** 1 Department of Healthcare Administration and Management, Graduate School of Healthcare Sciences, Kyushu University, Fukuoka, Japan; 2 Department of Preventive Medicine and Community Health, University of Occupational and Environmental Health, Fukuoka, Japan; 3 Department of Health Policy and Informatics, Tokyo Medical and Dental University, Graduate School of Medicine, Tokyo, Japan; 4 Department of Health Administration and Policy, Tohoku University, Miyagi, Japan; University of Kansas Medical Center, UNITED STATES

## Abstract

We examined the impact of opportunistic infections on in-hospital mortality, hospital length of stay (LOS), and the total cost (TC) among adult T-cell leukaemia (ATL) patients. In this retrospective cohort study, we identified 3712 patients with ATL using national hospital administrative data. Analysed opportunistic infections included *Aspergillus* spp., *Candida* spp., cytomegalovirus (CMV), herpes simplex virus (HSV), pneumocystis pneumonia (PCP), tuberculosis, varicella zoster virus (VZV), *Cryptococcus* spp., nontuberculous mycobacteria, and *Strongyloides* spp. Multilevel logistic regression analysis for in-hospital mortality and a multilevel linear regression analysis for LOS and TC were employed to determine the impact of opportunistic infections on clinical outcomes and healthcare resources. We found ATL patients infected with CMV had significantly higher in-hospital mortality (adjusted odds ratio (AOR) 2.29 [1.50–3.49] *p* < 0.001), longer LOS (coefficient (B): 0.13 [0.06–0.20] *p* < 0.001) and higher TC (B: 0.25 [0.17–0.32] *p* < 0.001) than those without CMV. Those with CAN and PCP were associated with a lower in-hospital mortality rate (AOR 0.72 [0.53–0.98] *p* = 0.035 and 0.54[0.41–0.73] *p* < 0.001, respectively) than their infections. VZV was associated with longer LOS (B: 0.13 [0.06–0.19] *p* < 0.001), while aspergillosis, HSV, or VZV infections were associated with higher TC (B: 0.16 [0.07–0.24] *p* < 0.001, 0.12 [0.02–0.23] *p* = 0.025, and 0.17 [0.10–0.24] *p* < 0.001, respectively). Our findings reveal that CMV infection is a major determinant of poor prognosis in patients affected by ATL.

## Introduction

Adult T-cell leukaemia (ATL) is a peripheral T cell neoplasm that is characterized by monoclonal proliferation of T cells infected by human T-lymphotrophic virus (HTLV-1) [1]. HTLV-1 is endemic in southwestern Japan, Africa, the Caribbean Islands, and South America [2]. Estimated prevalence and annual incidence rates of ATL in Japan are 1.2 million and 800, respectively [3]. Shimoyama classification of ATL constitutes four clinical subtypes: acute, lymphoma, chronic, and smouldering type [4]. The median survival time ranges from approximately 3 to 6 months for acute and lymphoma types, while the median survival is 2 years or more for indolent smouldering and chronic types [5]. Immediate conventional chemotherapy is the recommended first-line treatment for ATL, while allo-hematopoietic stem-cell transplantation (allo-HSCT) may be considered a treatment option for some patients [1]. Although allo-HSCT can provide long-term remission [6], treatment availability is limited as it is only indicated for younger patients who have achieved sufficient disease control, and that have an appropriate stem cell source [7]. As such, most patients with ATL who are only treated with conventional chemotherapy have poor prognoses. With low response rates and lack of long-term efficacy, the longest reported median survival time by conventional chemotherapy is approximately 13 months [8].

ATL is further complicated by various opportunistic infections (OI), even in patients who do not undergo HSCT. These include pneumocystis pneumonia (PCP), mycoses, cytomegalovirus (CMV) infection, and strongyloidiasis [1]. Furthermore, CMV infections [9–12].PCP [13], and mycosis[14–16] are associated with poor prognoses in patients undergoing HSCT or solid organ transplantation. It has also been reported that patients taking immunosuppressive medications and that have OI use more health care resources[17–18] The aim of this study was to investigate the effects of OI on clinical outcomes in ATL patients who do not receive allo-HSCT, as well as associated healthcare resources.

## Materials and Methods

### Patients

A retrospective analysis of nationwide, electronic hospital administrative data that were mandatorily submitted by health care providers to Japan’s Ministry of Health, Labour, and Welfare as part of the Diagnostic-Procedure Combination/Per Diem Payment System (DPC/PDPS) was performed. As more than 90% of acute in-patient services are covered by DPC in Japan [19], these DPC data comprise anonymous charges, clinical, and care information [20]. The study included data submitted from June 1st, 2007 to November 30, 2009, and from June 1, 2010 to December 31, 2010. All data pertaining to patients aged 20 years or older diagnosed with ATL according to ‘C915’ in the *International Classification of Diseases*, *10* ^*th*^ *Revision* (ICD10), were extracted. At first, 4550 patients were identified. Of these, 176 patients who underwent HSCT (procedure code 'K922') or that were classified as post-HSCT (ICD10 codes ‘Z948’, 'T86') were excluded, as were 680 patients that had any missing data during this study period. Finally, a total of 3712 patients comprised the retrospectively analysed cohort.

### Study design

This retrospective cohort analysis closely followed internationally recognized Strengthening the Reporting of Observational Studies in Epidemiology (STROBE) guidelines [21]. The ethics committee at the University of Occupational and Environmental Health in Kitakyushu, Fukuoka, Japan, approved the study. Informed consent was not obtained because patient information was anonymized prior to analysis.

### Definition of OI

To define the OI examined in the study cohort, we identified ATL patients who were diagnosed with ‘A00’ to ‘B99’ in the ICD10. These codes refer to ‘certain infectious and parasitic diseases’. We focused on OI that were included in guidelines created by the US Centers for Disease Control and Prevention, the National Institute of Health, and the HIV Medicine Association of the Infectious Diseases Society of America [22]. OI for which a specific causative species was not identified (e.g. ‘fungal infection’ or ‘viral infection’) were excluded. Thus, the final list of OI in ATL patients that was examined in the present study included: *Aspergillus* spp. (ASP), *Candida* spp. (CAN), CMV, herpes simplex virus (HSV), PCP, tuberculosis (Tb), varicella zoster virus (VZV), *Cryptococcus* spp., nontuberculous mycobacteria (NTM), and *Strongyloides* spp.

### Definition of outcome variables

The outcome measures of this analysis were in-hospital mortality, hospital length of stay (LOS), and the total cost (TC) of healthcare during hospitalization. In-hospital mortality was defined as any mortality that occurred during hospitalization. TC was calculated in Japanese Yen (JPY; at 100 JPY per US dollar), as was billed during hospitalization, and this was used as a proxy for cost as described previously [23]. In Japan, hospital charges are determined by the national uniform fee schedule. In this study, TC included physician fees, instrument costs, laboratory or imaging test costs, and administration fees [23].

### Definition of other variables

Patients were categorized into three groups according to age: < 65 years, ≥ 65 and < 75 years, and ≥75 years. Hospital locations were categorized as ‘Kyushu district’ or ‘other than Kyushu district’, as the Kyushu district reportedly includes the most prevalent area of ATL in Japan [2]. Hospital admission course was categorized as planned or urgent admission. Comorbidities other than hematologic malignancy were identified by applying the Charlson comorbidity index (CCI) [24]. Comorbidities examined in this study included acquired immunodeficiency syndrome (AIDS), cardiovascular disease (CAD), cerebrovascular disease (CVD), mild diabetes, diabetes with complications, liver disease, primary malignancy, metastatic malignancy, and renal disease. Hypercalcaemia (‘E835’ in ICD10) and hypoalbuminaemia (‘E880’ in ICD10, or 'E43', 'E44', and 'E46' corresponding to ‘malnutrition’) during hospitalization were also included as they are often associated with ATL disease progression and prognosis [25,26]. Sepsis was considered another comorbidity as it often influences the clinical course of ATL [27].

All chemotherapeutic agents analysed in this study are described in the Japanese uniform fee schedule [28]. They include alkylating agents, antimetabolic agents, antitumor antibiotics, platinums, vinca alkaloids, taxane, and topoisomerase II inhibitors. Chemotherapeutic administration routes were categorized as oral or intravenous. Intravenous chemotherapy was further categorized as a single or multi-agent treatment. Although steroid use is a well-recognized risk factor for OI [28], it was excluded from our analysis because of multi-collinearity.

### Statistical analysis

Median and interquartile ranges were used for continuous variables and frequency and proportion were used for categorical variables in all descriptive analyses. For statistical analyses, the Mann-Whitney U and Kruskal-Wallis tests were performed for continuous variables and the chi-square test was performed for categorical variables. Associations between age groups, outcome, and other variables were also analysed using the chi-square test.

At first, univariate analyses were used to analyse the effects of OI on in-hospital mortality, LOS, and TC. These were followed by a multilevel logistic regression analysis for in-hospital mortality and a multilevel linear regression analysis for LOS and TC, with a random intercept model to differentiate data years. The forced entry method was employed with the outcome variables in-hospital mortality, LOS, and TC. We employed explanatory variables including gender, age groups, comorbidities, treatment, other clinical events, admission course, hospital district, and OI. The multilevel logistic regression model data are reported as odds ratios, while of the multilevel linear regression model data are reported as a regression coefficient (B).

We employed common logarithm transformation for LOS and TC, as both were right-skewed. We used log likelihood for goodness of fit in each model. Furthermore, we confirmed there was no multi-collinearly among variables in the multivariate regression analyses.

All statistical analyses were performed using Stata 13 software (Stata Corp., College Station, TX, USA). The ‘melogit’ Stata command was used for multilevel logistic regression analyses and the ‘mixed’ command was used for multilevel linear regression analyses. All reported *p* values were two-tailed, and statistical significance was set at *p* = 0.05.

## Results

### OI prevalence in ATL patients

The prevalence of OI of the retrospectively analysed ATL patient cohort is shown in Table 1. Approximately 31.1% (1154/3712) of the cohort had at least one infection. Specific infections diagnosed in more than 10% of the cohort included PCP (13.7%) and CAN (11.0%). The prevalence of the remaining OI that were examined was the following: Tb (3.8%), VZV (3.2%), CMV (2.9%), ASP (2.2%), HSV53 (1.4%), *Cryptococcus* spp. (0.5%), NTM (0.1%), and *Strongyloides* spp. (0.1%).

**Table 1:** Prevalence of OI in adult T-cell leukaemia patients.

Pathogen	N	(%)
PCP	508	(13.7)
Candida	407	(11.0)
Tuberculosis	140	(3.8)
VZV	118	(3.2)
Cytomegalovirus	109	(2.9)
Aspergillus	81	(2.2)
HSV	53	(1.4)
Cryptococcus	17	(0.5)
NTM	3	(0.1)
Strongyloides	2	(0.1)
Any infection	1154	(31.1)

HSV: Herpes simplex virus, NTM: Nontuberculous mycobacteria; PCP: Pneumocystis pneumonia; VZV: Varicella zoster virus. Total number of patients: 3712.

### Patient characteristics

Table 2 lists patient characteristics according to age category. The median (interquartile range) age was 68 (15) years. The proportion of patients in the < 65 years, ≥ 65 and < 75 years, and ≥ 75 years categories was 39.7%, 34.5%, and 25.9%, respectively. The number of male patients tended to be higher in all age groups. The proportion of patients treated in the Kyushu hospital district was approximately 60%, and this proportion tended to increase as patients aged. Similarly, the proportion of patients who required urgent admission tended to be higher as patients’ ages increased. Patients with primary neoplasm, CAD, and CVD tended to be older. There were no significant relationships between age and sepsis, hypercalcaemia, or hypoalbuminaemia. Additionally, we observed that patients aged 75 years and older underwent less intravenous chemotherapy with both single and multi-agents. Conversely, patients aged less than 65 years were likely to have less orally administered chemotherapy than the other age groups.

**Table 2:** Patient characteristics categorized by age.

		Age groups^a^			Total	p
		<65	65≦<75	75≦		
		(N = 1472)	(N = 1279)	(N = 961)	(N = 3712)	
Gender						
Male	(%)	762(51.8)	709(55.4)	491(51.1)	1962(52.9)	0.07
Female	(%)	710(48.2)	570(44.6)	470(48.9)	1750(47.1)	
District						
Kyushu	(%)	753(51.2)	759(59.3)	669(69.6)	2181(58.8)	<0.001
Other than Kyushu	(%)	719(48.8)	520(40.7)	292(30.4)	1531(41.2)	
Years						
2007	(%)	444(30.2)	325(25.4)	237(24.7)	1006(27.1)	0.008
2008	(%)	335(22.8)	278(21.7)	233(24.2)	846(22.8)	
2009	(%)	286(19.4)	307(24.0)	216(22.5)	809(21.8)	
2010	(%)	407(27.6)	369(28.9)	275(28.6)	1051(28.3)	
Admission course						
Planned	(%)	1133(77.0)	1005(78.6)	608(63.3)	2670(71.9)	<0.001
Urgent	(%)	339(23.0)	350(27.4)	353(36.7)	1042(28.1)	
Comorbidities						
AIDS	(%)	2(0.1)	1(0.1)	0(0.0)	3(0.1)	0.514
Cardiovascular disease	(%)	75(5.1)	104(8.1)	99(10.3)	278(7.5)	<0.001
Cerebrovascular disease	(%)	19(1.3)	40(3.1)	45(4.7)	104(2.8)	<0.001
Mild diabetes	(%)	128(8.7)	142(11.1)	92(9.6)	362(9.8)	0.103
Diabetes with complication	(%)	14(1.0)	8(0.6)	11(1.1)	33(0.9)	0.41
Liver disease	(%)	15(1.0)	20(1.6)	11(1.1)	46(1.2)	0.416
Primary malignancy	(%)	60(4.1)	69(5.4)	62(6.5)	191(5.1)	0.031
Metastatic malignancy	(%)	77(5.2)	53(4.1)	43(4.5)	173(4.7)	0.383
Renal disease	(%)	10(0.7)	37(2.9)	19(2.0)	66(1.8)	<0.001
Other clinical events						
Sepsis	(%)	78(5.3)	71(5.6)	47(4.9)	196(5.3)	0.787
Hypercalcemia	(%)	144(9.8)	150(11.7)	102(10.6)	396(10.7)	0.256
Hypoalbminemia	(%)	18(1.2)	19(1.5)	14(1.5)	51(1.4)	0.813
Treatments						
Intravenous chemotherapy						
Single agent	(%)	89(6.0)	50(3.9)	31(3.2)	170(4.6)	0.002
Multiple agent	(%)	1027(69.8)	909(71.1)	501(52.1)	2437(65.7)	<0.001
Peroral chemotherapy	(%)	141(9.6)	222(17.4)	163(17.0)	526(14.2)	<0.001
Oppotunistic infections						
Aspergillus	(%)	40(2.7)	24(1.9)	17(1.8)	81(2.2)	0.192
Candida	(%)	164(11.1)	162(12.7)	81(8.4)	407(11.0)	0.006
Cytomegalovirus	(%)	36(2.4)	49(3.8)	24(2.5)	109(2.9)	0.064
Herpes simplex virus	(%)	32(2.2)	14(1.1)	7(0.7)	53(1.4)	0.006
Pneumocystis pneumonia	(%)	212(14.4)	185(14.5)	111(11.6)	508(13.7)	0.082
Tuberculosis	(%)	36(2.4)	51(4.0)	53(5.5)	140(3.8)	<0.001
Varicella zoster virus	(%)	52(3.5)	49(3.8)	17(1.8)	118(3.2)	0.014
Other organism	(%)	10(0.7)	5(0.4)	7(0.7)	22(0.6)	0.504
Outcome						
In-hospital mortality	(%)	238(16.2)	287(22.4)	286(29.8)	811(21.8)	<0.001
Care resource uses						
LOS median	(IQR)	30(37.0)	29(41.0)	24(34.0)	28(38.0)	0.001 ^b^
TC median (dollar)	(IQR)	11385.3(16673.0)	10467.8(16952.6)	8705.0(11391.2)	10297.9(15219.7)	<0.001^b^

AIDS: Acquired immunodeficiency syndrome; IQR: Interquartile range, LOS: Length of stay, TC: Total charge.

^a^ Median age (interquartile range): 68 (15)

^b^ Kruskal-Wallis test, all others by chi-square test

Interestingly, we observed significant relationships between specific age groups and different prevalence rates for OI such as CAN, HSV, Tb, and VZV. For instance, the number of patients infected with Tb tended to increase with age. Furthermore, there were significant relationships between age and the proportion of patients with CAD, CVD, primary neoplasm, and renal disease. Finally, in-hospital mortality increased with age. However, LOS and TC were significant different between age groups. Specifically, TC and LOS tended to be less in older patients.

### Univariate analyses of OI in ATL patients on in-hospital mortality and healthcare costs

Table 3 shows the impact of OI on clinical outcomes and health care resources as defined by hospital LOS and TC. Patients infected with CAN, CMV, or PCP had significant relationships with in-hospital mortality. Patients infected with CMV, PCP, or VZV were significantly associated with LOS. Patients affected by any other infection other than CAN or Tb were significantly associated with a TC.

**Table 3:** Univariate analyses of opportunistic infections on in-hospital mortality, length of stay, and total charge.

	In-hospital mortality			LOS			TC		
	N	(%)	p	Median	(IQR)	p	Median	(IQR)	p
Opportunistic infections									
Aspergillus	23	(28.4)	0.149	29	(42.5)	0.471	14765.0	(21529.2)	<0.001
Candida	61	(15.0)	<0.001	30	(31.0)	0.694	10699.0	(14172.2)	0.383
Cytomegalovirus	43	(39.4)	<0.001	41	(53.0)	<0.001	20549.2	(28179.8)	<0.001
Herpes simplex virus	8	(15.1)	0.231	35	(39.0)	0.151	14143.0	(20747.3)	0.011
Pneumocystis pneumonia	66	(13.0)	<0.001	30	(30.0)	0.027	10994.2	(14471.4)	0.002
Tuberculosis	22	(15.7)	0.073	29.5	(27.5)	0.767	12033.0	(11713.2)	0.262
Varicella zoster virus	30	(25.4)	0.339	37	(51.3)	0.002	15382.6	(20933.9)	<0.001
Other organism	10	(45.5)	0.007	43	(60.0)	0.012	25154.2	(29231.6)	0.001

In-hospital mortality analysed by chi-square test, LOS and TC by Mann–Whitney U test. IQR: Interquartile range; LOS: Length of stay (days); TC: Total charges (US dollars).

### Multivariate analyses of OI and other confounding factors on in-hospital mortality and health care costs

Table 4 summarizes the impact of OI and other confounding factors on in-hospital mortality and healthcare resource as defined by LOS and TC. Patients infected with CMV had a significantly higher in-hospital mortality rate than patients who were not infected with CMV (Adjusted odds ratio (AOR) in-hospital mortality 2.29 [1.50–3.49] *p* < 0.001). To the contrary, patients infected with CAN or PCP had significantly lower in-hospital mortality rates than patients who did not have their infections (AOR 0.72 [0.53–0.98] *p* = 0.035 for CAN and 0.54 [0.41–0.73] *p* < 0.001 for PCP). When LOS was examined, patients infected with CMV or VZV had significantly longer LOS (coefficient (B): 0.13 [0.06–0.20] *p* < 0.001 in CMV and 0.13 [0.06–0.19] *p* < 0.001 in VZV) than patients that were not infected with their infections. For TC, patients with an infection other than PCP, CAN, or Tb had significantly higher TC (B: 0.16 [0.07–0.24] *p* < 0.001 in ASP, 0.25 [0.17–0.32] *p* < 0.001 in CMV, 0.12 [0.02–0.23] *p* = 0.025 in HSV, 0.17 [0.10–0.24] *p* < 0.001 in VZV, and 0.20 [0.04–0.37] *p* = 0.015 in other organism). The effect of other confounding factors on in-hospital mortality and healthcare resources as determined by multivariate analyses are also shown in Table 4.

**Table 4:** Multivariate analyses of OI and other factors on in-hospital mortality and health care costs.

	In-hospital mortality			LogLOS			LogTC		
	AOR	[95%CI]	p	B	[95%CI]	p	B	[95%CI]	p
Opportunistic infections									
Aspergillus	1.46	[0.85-2.52]	0.168	0.03	[-0.05-0.11]	0.474	0.16	[0.07-0.24]	<0.001
Candida	0.72	[0.53-0.98]	0.035	-0.01	[-0.05-0.03]	0.641	-0.01	[-0.05-0.03]	0.694
Cytomegalovirus	2.29	[1.50-3.49]	<0.001	0.13	[0.06-0.20]	<0.001	0.25	[0.17-0.32]	<0.001
Herpes simplex virus	0.86	[0.39-1.91]	0.711	0.05	[-0.05-0.15]	0.311	0.12	[0.02-0.23]	0.025
Pneumocystis pneumonia	0.54	[0.41-0.73]	<0.001	0.01	[-0.03-0.04]	0.739	0.01	[-0.03-0.05]	0.625
Tuberculosis	0.89	[0.54-1.46]	0.651	0.01	[-0.05-0.08]	0.744	0.06	[-0.01-0.12]	0.105
Varicella zoster virus	1.32	[0.84-2.09]	0.228	0.13	[0.06-0.19]	<0.001	0.17	[0.10-0.24]	<0.001
Other organism	1.97	[0.74-5.28]	0.175	0.13	[-0.03-0.29]	0.121	0.20	[0.04-0.37]	0.015
Gender									
Male	reference			reference			reference		
Female	0.85	[0.72-1.01]	0.065	0.00	[-0.02-0.03]	0.717	-0.01	[-0.03-0.02]	0.582
Ages									
<65	reference			reference			reference		
65≦ <75	1.44	[1.17-1.77]	0.001	-0.02	[-0.05-0.01]	0.123	-0.04	[-0.07--0.01]	0.006
75≦	1.95	[1.56-2.42]	<0.001	-0.01	[-0.04-0.02]	0.616	-0.06	[-0.09--0.03]	<0.001
District									
Kyushu	1.06	[0.89-1.27]	0.501	-0.06	[-0.08--0.03]	<0.001	-0.08	[-0.11–-0.06]	<0.001
Other than Kyushu	reference			reference			reference		
Admission course									
Urgent	3.00	[2.49-3.60]	<0.001	0.09	[0.06-0.12]	<0.001	0.13	[0.10-0.16]	<0.001
Planned	reference			reference			reference		
Comorbiditis									
AIDS	6.33	[0.43-92.35]	0.177	0.13	[-0.29-0.56]	0.542	0.18	[-0.26-0.62]	0.429
Cardiovascular disease	0.86	[0.62-1.21]	0.387	-0.02	[-0.07-0.03]	0.370	-0.02	[-0.07-0.03]	0.451
Cerebrovascular disease	1.39	[0.87-2.20]	0.166	0.03	[-0.04-0.11]	0.382	0.01	[-0.06-0.09]	0.715
Mild diabetes	0.75	[0.56-1.01]	0.061	0.01	[-0.03-0.05]	0.536	0.00	[-0.04-0.04]	0.946
Diabetes with complication	0.72	[0.29-1.79]	0.480	0.05	[-0.07-0.18]	0.406	0.04	[-0.09-0.18]	0.551
Liver disease	1.40	[0.71-2.77]	0.333	-0.03	[-0.14-0.08]	0.608	0.02	[-0.10-0.13]	0.759
Primary malignancy	0.68	[0.46-1.02]	0.063	0.11	[0.05-0.16]	<0.001	0.09	[0.04-0.15]	0.001
Metastatic malignancy	1.56	[1.08-2.25]	0.017	0.08	[0.03-0.14]	0.004	0.12	[0.06-0.18]	<0.001
Renal disease	1.98	[1.14-3.44]	0.015	0.00	[-0.09-0.10]	0.944	0.02	[-0.07-0.12]	0.635
Other clinical events									
Sepsis	2.15	[1.55-3.00]	<0.001	0.21	[0.16-0.27]	<0.001	0.28	[0.23-0.34]	<0.001
Hypercalcemia	1.68	[1.32-2.15]	<0.001	0.05	[0.01-0.09]	0.012	0.07	[0.03-0.12]	<0.001
Hypoalbminemia	5.84	[3.08-11.06]	<0.001	0.11	[0.00-0.21]	0.048	0.18	[0.07-0.29]	0.001
Treatments									
Intravenous chemotherapy									
Single agent	1.49	[1.01-2.19]	0.043	0.13	[0.07-0.19]	<0.001	0.16	[0.10-0.23]	<0.001
Multiple agent	0.86	[0.71-1.04]	0.117	0.35	[0.33-0.38]	<0.001	0.39	[0.36-0.42]	<0.001
Peroral chemotherapy	1.17	[0.93-1.47]	0.191	0.31	[0.27-0.34]	<0.001	0.27	[0.23-0.31]	<0.001

In-hospital mortality analysed by multilevel logistic regression model, log LOS and log TC by multilevel linear regression model, data year by random intercept. AOR: Adjusted odds ratio; AIDS: Acquired immunodeficiency syndrome; B: coefficient; LOS: Length of stay; TC: Total charge.

## Discussion

Published data are sparse on the clinical outcomes of ATL patients who become infected with an OI during their disease course. To enrich the literature, we retrospectively analysed 3712 adult Japanese patients with ATL that did not undergo HSCT to examine the effect of OI on patient outcomes and healthcare resources as defined by hospital LOS and TC.

First, we questioned which type of infection was a major determining factor for in-hospital mortality and increased use of healthcare resources. We found that CMV was significantly associated with a higher in-hospital mortality rate, longer LOS, and higher TC. In addition, VZV was significantly associated with a longer LOS, while ASP, HSV, and VZV were significantly associated with higher TC.

CMV is a ubiquitous herpesvirus that infects 40–100% of adults; clinical presentation of an active CMV infection varies and could include pneumonitis, hepatitis, colitis, and retinitis [9]. Although primary CMV infection is usually latent, it could be fatal for immunocompromised individuals such as those living with AIDS or taking immunosuppressive medications. It is well established that CMV is prevalent among ATL patients, even in individuals that have not undergone transplantation [29, 30]. However, prognoses for ATL patients that do not undergo HSCT with CMV have been differentially reported [29,31,32]. It was first reported that CMV infection is a significant predictive factor for both poor prognosis and higher healthcare resources use among ATL patients without HSCT. Our findings support previous studies demonstrating that CMV infection is a major cause of mortality and morbidity among patients who take immunosuppressive medications [9–12]. It is probable that a poor prognosis in CMV-infected ATL patients is not only due to the direct consequences of CMV such as pneumonitis [29], but also superinfection caused by CMV immunosuppression[10,32].

Interestingly, we found that patients infected with PCP or CAN had significantly lower in-hospital mortality rates than those without their infections, despite being previously associated with poor prognoses for patients who underwent HSCT [14–16,33]. One possible explanation is that the immune capacities of patients infected with PCP or CAN are not impaired deeply. According to guidelines created by US Centers for Disease Control and prevention, the National Institutes of Health, and the HIV Medicine Association of the Infectious Diseases, CAN or PCP occur in HIV-infected patients when CD4+ counts < 200 cells/μL; in contrast, CMV typically occurs when CD4+ counts < 50 cells/μL [22]. Thus, ATL patients infected with CAN or PCP that may respond better to treatment as they relatively reserve immune capacities, whereas patients with CMV infection may be severely immunocompromised and this could lead to superinfection. In addition, as information and established effective treatments on these infections have been increasingly available to among healthcare providers in Japan, it could have contributed to improvement in mortality.

Our analysis also determined that CMV or VZV infections were significantly associated with longer LOS. This might be due to various comorbidities that occur with either of these infections. For instance, it has been reported that post-herpetic neuralgia and bacterial skin infections associated with VZV [34] are more severe among immunosuppressed patients [35]. Similarly, CMV, VZV, HSV, or ASP infections were associated with a higher TC, while patients infected with CMV or VZV had a higher TC because of various comorbidities and a longer LOS in this study cohort. HSV or ASP, however, was not associated with a longer LOS. This is most likely because recommended medications such as acyclovir and valacyclovir for HSV [22], and voriconazole, amphotericin B, and caspofungin [22] for ASP are expensive [36], as are medications recommended for CMV and VZV.

Our results further demonstrate that patients with CMV infection have a higher in-hospital mortality rate. Together our data indicate that effective management of CMV infection is essential for improving clinical outcomes in these patients. However, there is very little reported evidence on efficient CMV therapy, including optimal timing of initiation of treatment in patients who will not undergo HSCT. Prophylaxis or pre-emptive therapy for CMV has been recommended for allo-HSCT ATL patients [37], as CMV pneumonitis can be fatal with a reported mortality rate is approximately 30–50% [9]. We suggest that recommendations for patients who will not undergo allo-HCST should be different than recommendations for patients undergoing allo-HSCT, as these patients’ immune systems can be revitalized if disease aggregation is controlled.

The strength of the present study lies in the use of a large-scale national data set that allowed for an abundant sample size. We concede, however, that the DPC data were extracted per episode and not per person. Therefore, we were unable to analyse clinical information such as performance status and laboratory data. In addition, we could not identify the ATL subtype for each patient by hospital administrative data alone; this was a critical study limitation. Nevertheless, information gained on the impact of OI on ATL patient outcomes and healthcare resources underlines the need for improved management strategies for affected individuals.

In conclusion, CMV infection is a major determinant of poor prognosis in adult ATL patients. It is associated with a higher in-hospital mortality rate, longer LOS, and higher TC. Further studies are needed to examine how management strategies for ATL patients infected with CMV can be improved.
